# Propagation Speeds of Relativistic Conformal Particles from a Generalized Relaxation Time Approximation

**DOI:** 10.3390/e26110927

**Published:** 2024-10-30

**Authors:** Alejandra Kandus, Esteban Calzetta

**Affiliations:** 1 Departamento de Ciências Exatas, Universidade Estadual de Santa Cruz, Rodov. J. Amado km 16, Salobrinho, Ilhéus 45662-900, BA, Brazil; kandus@uesc.br; 2 Departamento de Física, Facultad de Ciencias Exactas y Naturales, Universidad de Buenos Aires, Ciudad Universitaria, Ciudad de Buenos Aires CP 1428, Argentina; 3 Instituto de Física de Buenos Aires (IFIBA), y CONICET—Universidad de Buenos Aires, Ciudad de Buenos Aires CP 1428, Argentina

**Keywords:** relativistic hydrodynamics, relativistic kinetic theory, propagation speeds

## Abstract

The propagation speeds of excitations are a crucial input in the modeling of interacting systems of particles. In this paper, we assume the microscopic physics is described by a kinetic theory for massless particles, which is approximated by a generalized relaxation time approximation (RTA) where the relaxation time depends on the energy of the particles involved. We seek a solution of the kinetic equation by assuming a parameterized one-particle distribution function (1-pdf) which generalizes the Chapman–Enskog (Ch-En) solution to the RTA. If developed to all orders, this would yield an asymptotic solution to the kinetic equation; we restrict ourselves to an approximate solution by truncating the Ch-En series to the second order. Our generalized Ch-En solution contains undetermined space-time-dependent parameters, and we derive a set of dynamical equations for them by applying the moments method. We check that these dynamical equations lead to energy–momentum conservation and positive entropy production. Finally, we compute the propagation speeds for fluctuations away from equilibrium from the linearized form of the dynamical equations. Considering relaxation times of the form $\tau =\tau_{0}{\left(-\beta_{\mu }p^{\mu }\right)}^{-a}$, with −∞<a<2, where $\beta_{\mu }=u_{\mu }/T$ is the temperature vector in the Landau frame, we show that the Anderson–Witting prescription a=1 yields the fastest speed in all scalar, vector and tensor sectors. This fact ought to be taken into consideration when choosing the best macroscopic description for a given physical system.

## 1. Introduction

The success of hydrodynamics in the description of the early stages of relativistic heavy ion collisions [1,2] and the promise of relevant cosmological applications [3,4,5] have turned the study of strongly interacting systems of relativistic particles into an active area of research [6,7,8]. Although kinetic theory provides the microscopic description for these systems [9,10,11,12], the full Boltzmann equations are generally regarded as too complex, and simpler schemes consistent with the most important physical features are sought. Among these simpler schemes, the Relaxation Time Approximation (RTA), which assumes a collision term parameterized by a relaxation time, stands out. The RTA includes the nonlinearities in the theory through the equilibrium of one particle distribution function (1-pdf) towards which the system relaxes, and in this sense, the RTA ansatz contains the footprint of the nonlinear and complete underlying kinetic theory that describes the microphysics.

The first implementations of Bhatnagar, Gross and Krook’s (BGK) RTA [13] to relativistic fluids are those of Marle [14,15] and Anderson and Witting (AW) [16,17]. Both expressions are phenomenological ansatzes, proportional to $\left(f-f_{0}\right)$ and in each case, $f_{0}$ corresponds to different “frames” (see Section 3.3 below). In the case of Marle, the proportionality factor is m/τ, with *m* the mass of the gas particles, while in that of AW, it is $u_{\mu }p^{\mu }/\tau$. In both cases, the parameter τ is identified with a “relaxation time”. In principle the choice of the proportionality factor must be guided by phenomenological considerations or else by a systematic derivation form the Boltzmann equation, which is feasible in some cases; see refs. [18,19]. However successful, both Marle and AW’s RTAs severely distort the structure of the linearized Boltzmann equation, and their validity is doubtful for *soft* collision terms, where the continuous spectrum of the linearized collision operator reaches up to the zero eigenvalue associated with the hydrodynamic modes [20,21,22].

This has led to more general implementations of the RTA [23,24], where different modes are allowed to relax at different rates. However, simply allowing the relaxation times to be a function of energy other than constant or linear is not satisfactory as it may violate the energy–momentum conservation [25,26]. Rather, the RTA must be implemented while preserving the Hilbert space structure of the space of linearized one-particle distribution functions (1-pdfs). A kinetic equation allowing for a momentum-dependent relaxation time consistent with the energy–momentum conservation was introduced in [27,28]. In this paper, we elaborate on this proposal. In particular, we show how to produce an RTA matching any prescribed spectrum for the linearized collision operator, either soft or hard. Not being able to do this is one of the main drawbacks of the usual formulations of the RTA; see also [29,30,31,32,33,34,35,36,37,38].

Although a general solution of the RTA kinetic equation may be attempted, this leads to an integral equation for the equilibrium 1-pdf, which must be related to the actual 1-pdf through some prescription, which will be different for different choices of the relaxation time. For example, under the Marle prescription, the equilibrium 1-pdf leads to the same particle current as the actual one, while under the Anderson–Witting prescription, the equilibrium and actual 1-pdfs are matched though the energy current. This obscures the physical features of the system, which close enough to equilibrium is dominated by the hydrodynamic modes and a few long-lived non-hydrodynamic modes. To capture this behavior, it is best to assume a parameterized form for the 1-pdf, supplemented by dynamical equations for the parameters. In this paper, we obtain these dynamical equations by taking moments of the kinetic equation.

Of course, this poses the challenge of finding a suitable parameterization for the 1-pdf. The parameterization should be general enough to allow for an accurate description of physically meaningful processes but not so general as to make the ensuing theory unwieldly. Many proposals have been advanced in the literature [39,40,41,42,43,44].

In this paper, we adopt the point of view that the parameterization must include the Chapman–Enskog (Ch-En) solution to the kinetic equation as a particular case [45]. In other words, we use the second order Ch-En as a template, generalizing it to obtain a family of parameterized 1-pdfs, still containing the actual Ch-En solution as a particular case.

One reason for working this way is the fact that in the Ch-En expansion, as in DNMR [46] and IReD [47], each order is determined by the power of a certain small parameter. Thus, it is easy to identify the intensity of the deviation from the local thermal balance.

The procedure we propose yields parameterizations with an increasing complexity depending on which order the Ch-En solution is computed to. In this manuscript we work to the second order [48,49,50], which in the AW case returns the theory already analyzed in [51].

Once the parameterization has been chosen, the next step is to find equations of motion for the parameters. We require that these equations both conserve energy and momentum and enforce the Second Law. Note that even if the kinetic theory allows for an *H*-theorem, a positive entropy production in the parameterized theory does not follow automatically, because the parameterized 1-pdf is not a solution of the kinetic equation. As shown in [52], a suitable set of equations of motion is derived by taking the moments of the kinetic equation against the same irreducible phase-space functions which appear in the parameterization, see below, Equation (17). Observe that in principle, this method does not require linearization on deviations from equilibrium (see also [53]), though in general, the resulting theory is too complex unless severely restricted by symmetry considerations.

The ultimate goal of this paper is to compute the propagation speeds for collective modes of a conformal real relativistic system of interacting particles [54,55,56,57,58,59]. We define the propagation speed as the velocity of a front across which the parameters are continuous, but their first derivatives are not. To derive the hydrodynamic equations, we apply the moments method to a kinetic equation under the RTA. We work in the Landau frame of the fluid and generalize the usual RTA by allowing an arbitrary dependence of relaxation time on the particle energy [27,28]. Different choices for this dependence mean different underlying kinetic theories or equivalently, microphysics.

One crucial step in this procedure is the choice of which moments of the 1-pdf are considered. As mentioned above, we adopt the criterion of choosing the functions of momentum which yield a second-order Chapman–Enskog solution. In other words, we work within a restricted class of 1-pdfs which generalizes the second-order Chapman–Enskog solution, retaining it as a particular case. This choice leads us to parameterize the 1-pdf in terms of functions of momentum which themselves depend on the way the relaxation time relates to energy, see Equation (65) below. Because of this, different choices of the functional dependence of the relaxation time lead to different dynamical equations for the system, not only in the terms which derive from the moments of the collision integral, where the dependence on the relaxation time is explicit, but also in the derivative terms. We thus obtain a family of dynamical theories with different phenomenology according to the choice of the relaxation time as a function of energy. We can understand this dependence as the remaining footprint of a nonlinear and complete kinetic theory, which is approximated by a mathematically more tractable RTA.

In summary, assuming the relaxation time dependent upon energy [25,26,27,28] we show, on one hand, how to compare the predictions from different choices of this dependence, and on the other hand, that those different choices lead to macroscopic models that are clearly distinguishable (in this case by producing different propagation speeds).

The propagation speeds of a theory are of course fundamental to determining causality. The theory we are considering here is thermodynamically stable by construction, and our results confirm the expectation that it is causal as well [60,61,62,63,64,65,66].

Propagation speeds are also relevant for the discussion of shocks [54,67,68]. The propagation speed in kinetic theory is the velocity of the fastest particle for which the 1-pdf is not zero, so it can be arbitrarily close to the speed of light *c* for a suitable 1-pdf [69]. There are also examples from field theory where the propagation speed is arbitrarily close to that of light [70]. Hydrodynamics, on the other hand, usually has a fastest propagation speed which is less than *c* by a finite amount [58]. For this reason, strong enough shocks in hydrodynamics are discontinuous. This discontinuity is not observed in kinetic theory and may be regarded as an artifact of the hydrodynamic approximation. When considering approximations to the full kinetic equations, as in this paper, the issue of which setup yields the fastest speed becomes most relevant, as this is also the framework which provides the best description of shocks.

To make the discussion more concrete, we consider a particular family of generalized RTAs where the relaxation time takes the form $\tau =\tau_{0}{\left(-\beta_{\mu }p^{\mu }\right)}^{-a}$, with −∞<a<2, where $\beta_{\mu }=u_{\mu }/T$ is the temperature vector in the Landau frame of the fluid. This family covers both the case where hard modes thermalize faster than soft modes and the converse. It also contains Marle and AW’s RTAs as the a=0 and a=1 particular cases, respectively. The upper limit in *a* is necessary to avoid infrared divergences in the equations of motion.

We find that the AW choice a=1, where we recover the results of [51], yields the fastest speeds.

To summarize, the main results of this paper are (a) the construction of a generalized RTA designed to match the spectrum of any linearized kinetic equation, (b) the derivation of a parameterized theory which is causal and stable and enforces both energy–momentum conservation and positive entropy production, (c) the computation of the propagation speeds for scalar, vector and tensor perturbations away from equilibrium for a family of generalized RTAs containing Marle’s and AW’s as particular cases, and (d) the verification that AW’s RTA yields the fastest speed within this family.

This paper is organized as follows: In Section 2, we shortly review the features of kinetic theory, and in Section 3, we elaborate on the generalized RTA as a substitute for the actual kinetic equation as derived from microphysics. In Section 4, we deduce the 1-pdf Ch-En solution to the Boltzmann equation up to the second order in gradients, for a momentum-dependent RTA. We then introduce a 1-pdf after the pattern of the Ch-En solution and derive the set of moment equations, which for the purposes of finding the propagation speeds may be particularized at the free-streaming regime. In Section 5, we perform a scalar–vector–tensor decomposition and write down the equation system corresponding to each sector. To have a glimpse of their solutions, we consider the family of RTA’s given by $\tau =\tau_{0}{\left(-\beta_{\mu }p^{\mu }\right)}^{-a}$, with −∞<a≤2 to avoid infrared divergences. In Section 6, we summarize the main conclusions. Details of the derivation of the Ch-En solution are given in Appendix A.

We work with natural units $\hbar =c=k_{B}=1$ and signature $\left(-,+,+,+\right)$.

## 2. Relativistic Kinetic Theory

The central object of a kinetic description is the one-particle distribution function (1-pdf), which gives the probability of finding a particle within a given phase-space cell, at a particular event and with a particular momentum, constrained to be on a mass shell and to have positive energy [71,72,73,74]. For simplicity, we consider only gases whose equilibrium distribution is of the Maxwell–Jüttner kind (Equation (2)). The 1-pdf is advected by the particles and changes because of collisions among particles. Therefore, the kinetic equation has a transport part and a collision integral which gives the change in the 1-pdf due to collisions per unit particle proper time, as in

(1) $$p^{\mu }\frac{\partial f}{\partial x^{\mu }}=I_{coll}$$

There is fairly universal agreement about the transport part, while different kinetic approaches posit different collisions operators [6]. A collision integral must be consistent with energy–momentum conservation (for simplicity, we deal below with massless particles, and thus we do not impose particle number conservation) and allow for an *H*-theorem, the entropy production being zero only for Maxwell–Jüttner 1-pdfs

(2) $$f_{0}=e^{\beta_{\mu }p^{\mu }}$$

where $\beta_{\mu }=u_{\mu }/T$, with *T* being the fluid temperature and $u_{\mu }$ the velocity in a frame to be chosen below, with $u^{2}=-1$. Then,

(3) $$I_{coll}\left[f_{0}\right]=0$$

The energy–momentum tensor (EMT) and entropy flux are

(4) $$\begin{array}{ccc} & & T^{\mu \nu }=\int Dp\;p^{\mu }p^{\nu }f \end{array}$$

(5) $$\begin{array}{ccc} & & S^{\mu }=\int Dp\;p^{\mu }f\left[1-\ln f\right] \end{array}$$

where

(6) $$Dp=2\frac{d^{4}p}{{\left(2\pi \right)}^{3}}\delta \left(-p^{2}\right)\theta \left(p^{0}\right)$$

is the Lorentz invariant momentum space volume element. Energy–momentum conservation then implies that

(7) $$\int Dp\;p^{\mu }I_{coll}=0$$

and the *H*-theorem

(8) $$-\int Dp\;\ln f\;I_{coll}\geq 0$$

for *any* function *f*. *If* *f* is a solution of the kinetic equation Equation (1), this leads to positive entropy production $S_{,\mu }^{\mu }\geq 0$.

The Landau–Lifshitz prescription provides a way to associate an inverse temperature vector to any 1-pdf, even if it is not of the Maxwell–Jüttner kind. Namely, we identify the four-velocity with the only timelike eigenvector of the EMT

(9) $$T_{\nu }^{\mu }u^{\nu }=-\rho u^{\mu }$$

whereby we identify ρ as the energy density, and then we derive a temperature from ρ by imposing the equilibrium dependence

(10) $$\rho =\frac{3}{\pi^{2}}T^{4}$$

Having identified $\beta^{\mu }$, we may build the corresponding Maxwell–Jüttner 1-pdf $f_{0}$, Equation (2). Moreover, the fact that lnf appears explicitly in the H-theorem Equation (8) suggests we decompose *f* as

(11) $$f=f_{0}e^{\chi }$$

Without loss of generality, we may also write

(12) $$I_{coll}\left[f\right]=f_{0}I_{coll}\left[\chi \right]$$

By virtue of Equation (7), $\ln f_{0}$ does not contribute to entropy creation, and then we find

(13) $$S_{;\mu }^{\mu }=-\int Dp\;f_{0}\;\chi \;I_{coll}\left[\chi \right]\geq 0$$

if *f* is a solution of the kinetic equation.

The ansatz Equation (11) guarantees a positive one-particle distribution function, even in a full nonlinear theory. Moreover, the entropy production Equation (13) already singles out the logarithm of the one-particle distribution function as playing a most important role. Using this feature, we introduce below an ansatz for the collision term (see Equation (34)) which leads to positive entropy production to all orders in deviations from equilibrium (see Equation (35)).

### Parameterized Kinetic Theory

Let us look for solutions of Equation (1) of the form

(14) $$f\equiv f_{hydro}=e^{\sum_{\alpha =0}^{n}C_{\alpha }X^{\alpha }}$$

In Equation (14), the $C_{\alpha }$, denote tensor fields in space-time, while the $X^{\alpha }$ are tensor fields in phase space. Note that α is not itself a tensorial index, it just numbers the different tensors in the theory. The contractions $C_{\alpha }X^{\alpha }$ are world scalars. In particular, we choose $X^{0}=p^{\mu }$ and $C_{0}=\beta_{\mu }$, or equivalently, in the terms of Equation (11)

(15) $$\chi =\sum_{\alpha =1}^{n}C_{\alpha }X^{\alpha }$$

We assume $C_{\alpha }\left(x\right)$ are totally symmetric, traceless and transverse tensors, for α≥1. Of course, if we allowed the $X^{\alpha }$ functions to form a complete set in phase space, we could seek an exact solution of the kinetic equations under this form. However, we truncate the sum in Equation (15) at a finite value of *n*, to be specified below, so we obtain only an approximate solution.

The EMT and entropy flux are obtained by substituting $f_{hydro}$ into Equations (4) and (5). We obtain the four conservation laws (7) but these equations are not enough to determine the evolution of the whole set of $C_{\alpha }$ functions, for which we must provide supplementary equations.

Our guiding principle is to obtain positive entropy production. Now,

(16) $$S_{hydro,\mu }^{\mu }=-\int \;Dp\;\left[\sum_{\alpha =1}^{n}C_{\alpha }X^{\alpha }\right]p^{\mu }f_{hydro,\mu }$$

The problem is that we cannot call upon the H-theorem to enforce positive entropy production, because $f_{hydro}$ is not a solution of the kinetic equation. We demand instead the moment equations

(17) $$\int Dp\;X^{\alpha }\left\{p^{\mu }f_{hydro,\mu }-f_{0}I_{coll}\left[\chi \right]\right\}=0$$

which for α=0 is just EMT conservation. We now are allowed to substitute

(18) $$S_{hydro,\mu }^{\mu }=-\int \;Dp\;f_{0}\chi I_{coll}\left[\chi \right]\geq 0$$

The moment Equation (17) are thus the equations of motion of the parameterized theory.

This setup enforces positive entropy production but does not tell us how to choose the $X^{\alpha }$ functions, beyond α=0. We return to this vexing question in Section 4, after we have introduced the relaxation time approximation.

## 3. Relaxation Time Approximation

Physically, the role of the collision term in the kinetic Equation (1) is to force χ to relax to zero, or at least to a multiple of $p^{\mu }$, the only possibilities leading to vanishing entropy production. A realistic kinetic equation such as Boltzmann’s typically leads to a very complex collision term. However, it may be expected that the essentials of the relaxation of χ may be captured by a much simpler collision term, linear in χ

(19) $$I_{coll}\left[\chi \right]\left(x,p\right)=\int Dp^{\prime }\;f_{0}\left(x,p^{\prime }\right)K\left[p,p^{\prime }\right]\chi \left(x,p^{\prime }\right)$$

The linearization of the Boltzmann collision term yields an operator *K* which is symmetric in the space of momentum functions with the inner product [20,21,75]

(20) $$\langle \chi^{\prime }|\chi \rangle =\int Dp\;f_{0}\chi^{\prime }\chi$$

namely,

(21) $$\int Dp\;f_{0}\chi^{\prime }I_{coll}\left[\chi \right]=\int Dp\;f_{0}\chi I_{coll}\left[\chi^{\prime }\right]$$

$I_{coll}\left[\chi \right]$ has exactly four null eigenvectors corresponding to the hydrodynamic modes $\chi_{\mu }=p_{\mu }$; this enforces energy–momentum conservation. Moreover, the *H*-theorem Equation (13) requires that all nonzero eigenvalues of the collision operator be negative.

We call a kinetic equation with a collision term as in Equation (19) a generalized relaxation time approximation. The first relativistic RTA was Marle’s [14,15], who wrote the collision operator of the form

(22) $$I_{coll}^{\left(M\right)}\left[f\right]=\frac{\left(-T\right)}{\tau }\left[f-f_{0}^{\left(M\right)}\right]$$

We must mention that Marle’s original expression has a mass *m* and not a temperature *T*. But as we work with massless particles, the only relevant dimensionful parameter is *T*. Energy–momentum conservation requires

(23) $$\int Dp\;p^{\mu }\left[f-f_{0}^{\left(M\right)}\right]=\int Dp\;p^{\mu }f-nu^{\mu }=0$$

where $n=T^{3}/\pi^{2}$ would be the particle density for massless particles. Thus, Marle’s equation requires us to work in the so-called Eckart frame: we identify the velocity and temperature by matching the particle current of the actual 1-pdf [76].

After Marle, Anderson and Witting [16,17] proposed

(24) $$I_{coll}^{\left(AW\right)}\left[f\right]=\frac{\left(u_{\nu }p^{\nu }\right)}{\tau }\left[f-f_{0}^{\left(AW\right)}\right]$$

so now

(25) $$\int Dp\;p^{\mu }u_{\nu }p^{\nu }\left[f-f_{0}^{\left(AW\right)}\right]=u_{\nu }T^{\mu \nu }+\rho u^{\mu }=0$$

where $\rho =3T^{4}/\pi^{2}$ is the energy density for a conformal fluid; we see that in the AW formulation, $f_{0}$ is the equilibrium solution in the Landau–Lifshitz frame [77]. Both Marle and AW’s choices seriously distort the Boltzmann dynamics and are actually disfavored by experimental data from relativistic heavy ion collisions [23,24].

### 3.1. Generalized Relaxation Time Approximation

Concretely, our concern is to go beyond the Marle and Anderson–Witting RTAs by allowing the relaxation time to depend on the energy of the particle in nontrivial ways. It is clearly seen that trying to improve on Marle or AW’s equations by allowing τ to be momentum-dependent, while keeping the Eckart or Landau–Lifshitz prescriptions to identify the inverse temperature vector, leads to a contradiction [25,26]. In this section, we review the collision term proposed in [27,28], which overcomes this difficulty. For simplicity we work in the Landau–Lifshitz frame throughout; this has the appealing feature that it may be determined from the properties of the macroscopic energy–momentum tensor alone.

We introduce the notation

(26) $$\langle X\rangle =\langle 1|X\rangle =\int Dp\;f_{0}\;X$$

where $f_{0}$ is the Maxwell–Jüttner distribution Equation (2) built from the Landau–Lifshitz temperature and velocity. We assume the constraint

(27) $$u_{\mu }\langle p^{\mu }p^{\nu }\chi \rangle =0$$

which follows from applying the Landau–Lifshitz prescription to a linear order in the deviation from equilibrium χ.

We write the collision integral as in Equation (12). Energy–momentum conservation requires $I_{coll}\left[\chi \right]$ to be orthogonal to the four null eigenvectors $p^{\mu }$. To satisfy this requirement, we introduce a projection operator *Q* such that for any *g*

(28) $$\langle p^{\mu }Q\left[g\right]\rangle =0$$

It is symmetric

(29) $$\langle g^{\prime }Q\left[g\right]\rangle =\langle gQ\left[g^{\prime }\right]\rangle$$

and

(30) $$Q\left[g\right]=g\;\;\;\Leftrightarrow \;\;\;\langle p^{\mu }g\rangle =0$$

These properties suggest that

(31) $$Q\left[g\right]=g-p^{\nu }T_{0\nu \rho }^{-1}\langle p^{\rho }g\rangle$$

with

(32) $$T_{0}^{\mu \nu }=\langle p^{\mu }p^{\nu }\rangle =\rho \left[u^{\mu }u^{\nu }+\frac{1}{3}\Delta^{\mu \nu }\right]$$

(where $\Delta^{\mu \nu }=\eta^{\mu \nu }+u^{\mu }u^{\nu }$) is the energy–momentum tensor built from $f_{0}$, and

(33) $$T_{0\mu \nu }^{-1}=\frac{1}{\rho }\left[u_{\mu }u_{\nu }+3\Delta_{\mu \nu }\right]$$

From $\langle p^{\mu }Q\left[g\right]\rangle =0$ and the symmetry of *Q*, we conclude that $\langle gQ\left[p^{\mu }\right]\rangle =0$, and since *g* is arbitrary, it must be $Q\left[p^{\mu }\right]=0$, which is easily verified explicitly. Conversely, if $Q\left[g\right]=0$, then $g=\delta \beta_{\mu }p^{\mu }$ for some momentum-independent coefficients $\delta \beta_{\mu }$. Finally, acting with *Q* on both sides of Equation (31), we see that $Q^{2}=Q$, so *Q* is indeed a projection.

We may now define the collision integral. To preserve the symmetry, we propose

(34) $$I_{coll}\left[\chi \right]=-\frac{T^{2}}{\varsigma }Q\left[FQ\left[\chi \right]\right]$$

where ς is a dimensionless relaxation time, and $F=F\left[-\beta_{\mu }p^{\mu }\right]$ is a dimensionless function. The entropy production Equation (13) becomes

(35) $$S_{,\mu }^{\mu }=\frac{T^{2}}{\varsigma }\langle F{\left(Q\left[\chi \right]\right)}^{2}\rangle$$

Therefore, the *H*-theorem requires F≥0.

The rationale of the proposed Equation (34) is to retain the fundamental features of the Boltzmann equation within a single mathematical structure yet keeping it flexible enough to accommodate phenomenological considerations. Among the former, the feature we want to keep is that the linearized Boltzmann operator is a symmetric operator in a certain Hilbert space [75], which may be therefore diagonalized, and whose spectrum bears basic information about the physics of the system, most notably whether we deal with a *hard* or *soft* collision term—in the former case, the ever-present zero eigenvalue is an isolated eigenvalue, while in the latter, it is part of the continuous spectrum [20,21,22]. We elaborate on this in Section 3.2. Note that the double projection operator *Q* in Equation (34) both makes the collision term symmetric and enforces energy momentum conservation within a single frame regardless of the function *F*. In this paper, we choose to work in the Landau frame throughout and expect to explore different frame choices in forthcoming work [78,79,80].

On the other hand, we leave a window open for phenomenology through the choice of the function *F* in Equation (34). By far the most common choices for *F* are Marle’s (F=constant) and Anderson–Witting’s ($F\propto -u_{\mu }p^{\mu }$), to be discussed in more detail below (Section 3.3). However, phenomenological considerations in the context of RHICs have led to the proposal of more general power laws [23,24], which in some cases may be systematically derived from the Boltzmann equation [18,19] and are actively under research [22,25,26,29,30,31,32,33,34,35,36,37,38]. In this paper, we only consider functions *F* defined through power laws; even within this restricted class we find propagation speeds are strongly dependent on the function *F*.

### 3.2. Spectral Considerations

Let us analyze the equation

(36) $$Q\left[g\right]=h$$

We have the integrability conditions $\langle p^{\mu }h\rangle =0$ (or else, $Q\left[h\right]=Q^{2}\left[g\right]=Q\left[g\right]=h$), so *h* itself is a particular solution. Since the $p^{\mu }$’s are homogeneous solutions (note that here, μ is not a world index, it simply distinguishes each of four different functions from each other), the general solution is

(37) $$g=h+c_{\mu }p^{\mu }$$

We may now analyze the spectrum of $I_{coll}$. Suppose

(38) $$Q\left[FQ\left[\zeta_{\lambda }\right]\right]=\lambda \zeta_{\lambda }$$

If λ=0, then $FQ\left[\zeta_{\lambda }\right]=c_{\mu }p^{\mu }$, and then

(39) $$Q\left[\zeta_{\lambda }\right]=\frac{c_{\mu }p^{\mu }}{F}$$

Therefore, we must have the integrability condition

(40) $$c_{\nu }\langle \frac{p^{\mu }p^{\nu }}{F}\rangle =0$$

but this is impossible unless $c_{\mu }=0$ as well. Thus, we must have $Q\left[\zeta_{\lambda }\right]=0$. We conclude that the only null eigenvectors are indeed the $p^{\mu }$ functions.

Now, assume λ≠0. Then, $Q\left[\zeta_{\lambda }\right]=\zeta_{\lambda }$, and therefore, we may write

(41) $$Q\left[F\zeta_{\lambda }\right]=\lambda \zeta_{\lambda }$$

with general solution (see Equation (37))

(42) $$F\zeta_{\lambda }=\lambda \zeta_{\lambda }+c_{\mu }p^{\mu }$$

If $F\left[p^{\mu }\right]\neq \lambda$ for every $p^{\mu }$, then

(43) $$\zeta_{\lambda }=\frac{c_{\mu }p^{\mu }}{F-\lambda }$$

but this is not possible because it violates the integrability condition for Equation (41) $\langle p^{\mu }\zeta_{\lambda }\rangle =0$. Therefore, we conclude that the spectrum of the collision operator is included in the image of *F*.

Now, assume that $F\left[p^{\mu }\right]=\lambda$ for some $p_{\lambda }^{\mu }$. Let us work in the Landau–Lifshitz rest frame where $u^{\mu }=\left(1,0,0,0\right)$. Then, the solution to Equation (42) is

(44) $$\zeta_{\lambda }=f_{\lambda }\left[\frac{\vec{p}}{p}\right]\delta \left(F-\lambda \right)+PV\left\{\frac{c_{\mu }p^{\mu }}{F-\lambda }\right\}$$

for some function $f_{\lambda }$. We have two possibilities. If

(45) $$\langle p^{\mu }f_{\lambda }\left[\frac{\vec{p}}{p}\right]\delta \left(F-\lambda \right)\rangle =0$$

then from $\langle p^{\mu }\zeta_{\lambda }\rangle =0$, we conclude that the $c_{\mu }$ themselves are zero. Otherwise, we obtain a linear equation from which we determine the $c_{\mu }$ coefficients. Thus, we see that we may easily find a function *F* to match any preordained spectrum for the collision operator.

### 3.3. Marle and Anderson–Witting

To conclude this section, we discuss whether it is possible to regard Marle’s Equation (22) and AW’s Equation (24) as particular cases of the collision term Equation (34).

To make contact with AW’s Equation (24), we set $F=F^{\left(AW\right)}\left[x\right]=x$. Recall that since we are defining the velocity and temperature according to the Landau–Lifshitz prescription, when we split the 1-pdf as in Equation (11), Equation (27) follows, and we obtain $\langle p^{\mu }F\chi \rangle =-\beta_{\nu }\langle p^{\mu }p^{\nu }\chi \rangle =0$, so $Q\left[F\chi \right]=F\chi$.

Assume we also have $\langle p^{\rho }\chi \rangle =0$ besides Equation (27). Then, $Q\left[\chi \right]=\chi$, and so $Q\left[FQ\left[\chi \right]\right]=Q\left[F\chi \right]=F\chi$, yielding the AW RTA. In the following, we refer to $F\left[x\right]=x$ as the Anderson–Witting prescription.

When we implement Marle’s proposal, we must take into account that Marle’s fiducial equilibrium 1-pdf is built from matching the particle rather than the energy flux, namely,

(46) $$\int Dp\;p^{\mu }f_{0}^{\left(M\right)}=\frac{T^{\left(M\right)3}}{\pi^{2}}u^{\left(M\right)\mu }=\int Dp\;p^{\mu }f=\frac{T^{3}}{\pi^{2}}u^{\mu }+\langle p^{\mu }\chi \rangle$$

Therefore, writing $T^{\left(M\right)}=T\left(1+\delta T^{M}\right)$ and $u^{\left(M\right)\mu }=u^{\mu }+\delta u^{\left(M\right)\mu }$ (with $u_{\mu }\delta u^{\left(M\right)\mu }=0$), we obtain

(47) $$\begin{array}{ccc} \delta T^{M} & = & -\frac{\pi^{2}}{3T^{3}}u_{\mu }\langle p^{\mu }\chi \rangle \\ \delta u^{\left(M\right)\mu } & = & \frac{\pi^{2}}{T^{3}}\Delta_{\rho }^{\mu }\langle p^{\rho }\chi \rangle \end{array}$$

It follows that

(48) $$f_{0}^{\left(M\right)}=f_{0}\left[1+p^{\mu }T_{0\mu \rho }^{-1}\langle p^{\rho }\chi \rangle \right]$$

From Equation (31), we see that the Marle collision integral Equation (22) is just the collision integral from Equation (34) with F=1.

In the following section we implement this formalism to obtain a second-order theory of relativistic conformal fluids.

## 4. Hydrodynamics from the Second-Order Chapman–Enskog Solution

As mentioned in the Introduction, the first (and probably main) challenge in seeking a parameterized solution to the kinetic theory is to find a suitable parameterization of the kinetic 1-pdf. Our proposal is to use the second-order Ch-En solution as a template. This means we work out the second-order Ch-En solution and then write it as in Equation (14), thus identifying the $X^{\alpha }\left(x,p\right)$ functions. Of course, in the actual solution, these functions are multiplied by given coefficients built from $\beta^{\mu }$ and its derivatives. We later replace these coefficients by unknown functions $C_{\alpha }$ obeying the equations of motion (17), thus obtaining a parameterization that generalizes the Ch-En solution.

The Ch-En solution is a systematic expansion of the 1-pdf in powers of the dimensionless relaxation time ς introduced in Equation (34) [45]. We therefore have a hierarchy of solutions, depending on which order we extend the expansion to.

In the moments’ or grad approximation on the other side, there is no explicit small parameter with respect to which we can perform a perturbative expansion. Thus, it is not clear a priori how many moments of the 1-pdf must be included to describe a given departure from equilibrium. To circumvent this situation, in this paper, we take the point of view that the parameterized 1-pdf should take the form of Equations (14) and (15), where the $X_{n}$ are the same functions of momentum as they appear in a Ch-En solution at some given order.

We use a fiducial temperature $T_{0}$ to build explicit dimensionless quantities, namely, we define $t=T/T_{0}$, and similarly, we make all other quantities non-dimensional by dividing or multiplying by $T_{0}$ as required. For simplicity, we do not introduce new names for the dimensionless quantities. The dimensionless Boltzmann Equation (34) reads

(49) $$p^{\mu }\frac{\partial }{\partial x^{\mu }}f=-f_{0}\frac{Q\left[t^{2}F\left[-\beta_{\nu }p^{\nu }\right]Q\left[\chi \right]\right]}{\varsigma }$$

To implement a perturbative scheme, we expand

(50) $$\chi =\sum_{n=1}^{\infty }\varsigma^{n}\chi_{n}$$

and replacing into Equation (49), we obtain

(51) $$\varsigma p^{\mu }\left\{\left[p^{\nu }\beta_{\nu ,\mu }\left(1+\sum_{n=1}^{\infty }\varsigma^{n}\chi_{n}\right)\right]+\sum_{n=1}^{\infty }\varsigma^{n}\chi_{n,\mu }\right\}=-\sum_{n=1}^{\infty }\varsigma^{n}Q\left[t^{2}F\left[-\beta_{\nu }p^{\nu }\right]Q\left[\chi_{n}\right]\right]$$

where we have linearized the transport term, which is accurate enough for the discussion below.

The Ch-En procedure aims at obtaining a solution of this equation as a expansion in powers of ς. Space derivatives in the left-hand side of Equation (51) are considered to be of “zeroth order”, while time derivatives, defined as $\dot{X}=u^{\mu }X_{,\mu }$, have their own development in powers of ς

(52) $$\dot{X}=\sum_{n=0}^{\infty }\varsigma^{n}{\dot{X}}^{\left(n\right)}$$

Replacing in Equation (51) and matching powers of ς, we obtain

(53) $$\begin{array}{ccc} & - & p^{\mu }u_{\mu }\left\{p^{\nu }{\dot{\beta }}_{\nu }^{\left(n\right)}+\sum_{m=1}^{n}p^{\nu }{\dot{\beta }}_{\nu }^{n-m}\chi_{m}+\sum_{m=1}^{n}{\dot{\chi }}_{m}^{\left(n-m\right)}\right\}\\ & + & \delta_{n0}p^{\rho }\Delta_{\rho }^{\mu }p^{\nu }\beta_{\nu ,\mu }+p^{\rho }\Delta_{\rho }^{\mu }p^{\nu }\beta_{\nu ,\mu }\chi_{n}+p^{\rho }\Delta_{\rho }^{\mu }\chi_{n,\mu }\\ & = & -Q\left[t^{2}F\left[-\beta_{\nu }p^{\nu }\right]Q\left[\chi_{n+1}\right]\right] \end{array}$$

Because of the projector in the right-hand side, at each order, we have an integrability condition

(54) $$\begin{array}{ccc} 0 & = & -u_{\mu }\left\{\langle p^{\lambda }p^{\mu }p^{\nu }\rangle {\dot{\beta }}_{\nu }^{\left(n\right)}+\sum_{m=1}^{n}\langle p^{\lambda }p^{\mu }p^{\nu }\chi_{m}\rangle {\dot{\beta }}_{\nu }^{n-m}+\sum_{m=1}^{n}\langle p^{\lambda }p^{\mu }{\dot{\chi }}_{m}^{\left(n-m\right)}\rangle \right\}\\ & + & \delta_{n0}\Delta_{\rho }^{\mu }\langle p^{\lambda }p^{\rho }p^{\nu }\rangle \beta_{\nu ,\mu }+\Delta_{\rho }^{\mu }\langle p^{\lambda }p^{\rho }p^{\nu }\chi_{n}\rangle \beta_{\nu ,\mu }+\Delta_{\rho }^{\mu }\langle p^{\lambda }p^{\rho }\chi_{n,\mu }\rangle \end{array}$$

We shall simplify these expressions by further linearizing on the *t* and $u_{\mu }$ derivatives. Then, Equation (53) reduces to

(55) $$-p^{\mu }u_{\mu }\left\{p^{\nu }{\dot{\beta }}_{\nu }^{\left(n\right)}+\sum_{m=1}^{n}{\dot{\chi }}_{m}^{\left(n-m\right)}\right\}+\delta_{n0}p^{\rho }\Delta_{\rho }^{\mu }p^{\nu }\beta_{\nu ,\mu }+p^{\rho }\Delta_{\rho }^{\mu }\chi_{n,\mu }=-Q\left[t^{2}FQ\left[\chi_{n+1}\right]\right]$$

and Equation (54) yields

(56) $$0=-u_{\mu }\left\{\langle p^{\lambda }p^{\mu }p^{\nu }\rangle {\dot{\beta }}_{\nu }^{\left(n\right)}+\sum_{m=1}^{n}\langle p^{\lambda }p^{\mu }{\dot{\chi }}_{m}^{\left(n-m\right)}\rangle \right\}+\delta_{n0}\Delta_{\rho }^{\mu }\langle p^{\lambda }p^{\rho }p^{\nu }\rangle \beta_{\nu ,\mu }+\Delta_{\rho }^{\mu }\langle p^{\lambda }p^{\rho }\chi_{n,\mu }\rangle$$

Solving these equations (see Appendix A.1 and Appendix A.2), we find the first order

(57) $$\chi_{1}=-\frac{p^{\mu }p^{\rho }}{F\left[-\beta_{\nu }p^{\nu }\right]}\frac{\sigma_{\mu \rho }}{2t^{3}}$$

where $\sigma_{\mu \rho }$ is the shear tensor

(58) $$\sigma_{\mu \nu }=\Lambda_{\mu \nu }^{\alpha \beta }u_{\alpha ,\beta }$$

(59) $$\Lambda_{\mu \nu }^{\alpha \beta }=\left[\Delta_{\mu }^{\alpha }\Delta_{\nu }^{\beta }+\Delta_{\nu }^{\alpha }\Delta_{\mu }^{\beta }-\frac{2}{3}\Delta_{\mu \nu }\Delta^{\alpha \beta }\right]$$

If we were to stop at this order and take F=const. we would parameterize χ with just the function $X_{1}=\Lambda_{\rho \sigma }^{\mu \nu }p^{\rho }p^{\sigma }/const$. This would lead us to the Israel–Stewart theory, which is not satisfactory; in particular, it yields no dynamics for tensor modes [51]. This is one of the reasons why we go one order further:

(60) $$\begin{array}{ccc} \chi_{2} & = & \frac{1}{t^{2}}\Sigma_{\nu \mu \rho }\frac{p^{\nu }p^{\mu }p^{\rho }}{2t^{3}F^{2}}-\frac{1}{t^{3}}u_{\mu }t_{,\lambda \tau }\Lambda_{\rho \nu }^{\lambda \tau }\frac{p^{\nu }p^{\mu }p^{\rho }}{2t^{3}F^{2}}\\ & + & \frac{1}{5t^{3}}\sigma_{\nu ,\rho }^{\rho }p^{\nu }\left[\frac{D_{2}}{F}+\frac{\Theta_{4}^{1}}{\Theta_{3}^{0}}\frac{p^{\mu }u_{\mu }}{tF}+{\left(\frac{p^{\mu }u_{\mu }}{tF}\right)}^{2}-D_{3}\right] \end{array}$$

The tensor $\Sigma_{\sigma \rho \lambda }$ is found from the decomposition

(61) $$\begin{array}{ccc} \Delta_{\tau }^{\lambda }p^{\tau }p^{\sigma }p^{\rho }\sigma_{\sigma \rho ,\lambda } & = & p^{\lambda }p^{\sigma }p^{\rho }\Sigma_{\sigma \rho \lambda }+\frac{2}{5}{\left(p^{\mu }u_{\mu }\right)}^{2}p^{\sigma }\sigma_{\sigma ,\rho }^{\rho } \end{array}$$

where $\Sigma_{\sigma \rho \lambda }$ is transverse, symmetric and traceless,

(62) $$D_{2}=\frac{\Theta_{4}^{1}\Theta_{3}^{1}}{\Theta_{3}^{0}\Theta_{2}^{1}}-\frac{\Theta_{4}^{2}}{\Theta_{2}^{1}}$$

and

(63) $$D_{3}=\left[D_{2}\frac{\Theta_{3}^{1}}{\Theta_{3}^{0}}-{\left(\frac{\Theta_{4}^{1}}{\Theta_{3}^{0}}\right)}^{2}+\frac{\Theta_{5}^{2}}{\Theta_{3}^{0}}\right]$$

with the functions

(64) $$\Theta_{n}^{m}=\frac{1}{2\pi^{2}}\int du\;\frac{u^{n+1}}{F^{m}\left[u\right]}e^{-u}$$

We observe that the whole second line of Equation (60) vanishes when the Anderson–Witting collision term is chosen. This is to be expected because in this limit, these terms collapse into two terms proportional to $p^{\mu }$ and to $p^{\mu }/u_{\rho }p^{\rho }$, which do not show up when the Anderson–Witting prescription F[x]=x is used from scratch.

This concludes the construction of the second–order Ch-En solution. We now must use $\chi_{1}$ and $\chi_{2}$ as templates whereby to identify which functions of momentum to include into a general parameterization of χ.

### 4.1. Dynamics from the Moments Approach

Assuming a parameterization of the form Equation (15) begs the question of which functions $X^{\alpha }$ ought to be included. In search of guidance, we look at the functions of momentum which actually show up in the second-order Ch-En solution. From Equations (57) and (60), we see that the second-order χ may be regarded as a linear combination of four tensor fields

(65) $$\begin{array}{ccc} X^{1} & = & \frac{1}{2t^{2}F}\Lambda_{\lambda \tau }^{\mu \rho }p^{\lambda }p^{\tau }\\ X^{2} & = & \frac{1}{6t^{3}F^{2}}\Lambda_{\lambda \sigma \tau }^{\mu \nu \rho }p^{\lambda }p^{\sigma }p^{\tau }\\ X^{3} & = & \frac{1}{2t^{2}F}\Lambda_{\lambda \tau }^{\mu \rho }p^{\lambda }p^{\tau }\left[-\frac{u_{\mu }p^{\mu }}{tF}-\gamma \right]\\ X^{4} & = & \frac{1}{t}\Delta_{\mu }^{\nu }p^{\mu }\left[\frac{D_{2}}{F}+\frac{\Theta_{4}^{1}}{\Theta_{3}^{0}}\frac{p^{\mu }u_{\mu }}{tF}+{\left(\frac{p^{\mu }u_{\mu }}{tF}\right)}^{2}-D_{3}\right] \end{array}$$

where

(66) $$\gamma =\frac{\Theta_{6}^{3}}{\Theta_{5}^{2}}$$

and $\Lambda_{\lambda \sigma \tau }^{\mu \nu \rho }$ is the projection over transverse, totally symmetric and traceless third-order tensors. The coefficients of the linear combination are the four tensors $C_{\alpha }$ that represent the different parameters of the theory and have the same symmetry properties as the corresponding coefficients in Equations (57) and (60). The $X^{\alpha }$ tensors are totally symmetric, traceless and transverse with respect to the Landau–Lifshitz velocity, and moreover, they obey the orthogonality condition

(67) $$\langle p^{0}X^{\alpha }X^{\beta }\rangle \propto \delta^{\alpha \beta }$$

The equations of motion are the moment Equation (17), where $\chi =\sum_{\alpha =1}^{4}C_{\alpha }X^{\alpha }$, which is consistent with the constraint Equation (27), and $f_{0}=e^{\beta_{\mu }p^{\mu }}$. The equation corresponding to α=0 is just the energy–momentum conservation.

### 4.2. The Complete Set of Equations of Motion

As our ultimate goal is to compute propagation speeds, we develop here the linear form of the transport part of the equations in (17) for the different $C_{\alpha }$’s. We begin by writing

(68) $$f_{,\mu }=f_{0}\left(\beta_{\nu ,\mu }p^{\nu }+\sum_{\alpha =1}^{4}C_{\alpha ,\mu }X^{\alpha }\right)$$

The moments of the transport term include the energy–momentum conservation

(69) $$\langle p^{\lambda }p^{\mu }\left(\beta_{\nu ,\mu }p^{\nu }+\sum_{\alpha =1}^{4}C_{\alpha ,\mu }X^{\alpha }\right)\rangle =0$$

and the moment equations

(70) $$\langle X^{\gamma }p^{\mu }\left(\beta_{\nu ,\mu }p^{\nu }+\sum_{\alpha =1}^{4}C_{\alpha ,\mu }X^{\alpha }\right)\rangle =\ldots$$

with γ=1,…,4. The right-hand sides of Equation (70) are immaterial because they contain no derivative terms, while propagation speeds are defined by the principal terms in the equations [81].

Working in the rest frame where $u^{\mu }=\left(1,0,0,0\right)$ and setting t=1, we have

(71) $$\begin{array}{ccc} \langle p^{0}p^{\mu }p^{\nu }\rangle \beta_{\nu ,\mu }+\sum_{\alpha =1}^{4}\left[{\dot{C}}_{\alpha }\langle {\left(p^{0}\right)}^{2}X^{\alpha }\rangle +C_{\alpha ,i}\langle p^{i}p^{0}X^{\alpha }\rangle \right] & = & 0\\ \langle p^{j}p^{\mu }p^{\nu }\rangle \beta_{\nu ,\mu }+\sum_{\alpha =1}^{4}\left[{\dot{C}}_{\alpha }\langle p^{0}p^{j}X^{\alpha }\rangle +C_{\alpha ,i}\langle p^{i}p^{j}X^{\alpha }\rangle \right]=0\\ \langle X^{\gamma }p^{\mu }p^{\nu }\rangle \beta_{\nu ,\mu }+\sum_{\alpha =1}^{4}\left[{\dot{C}}_{\alpha }\langle X^{\gamma }p^{0}X^{\alpha }\rangle +C_{\alpha ,i}\langle X^{\gamma }p^{i}X^{\alpha }\rangle \right] & = & 0 \end{array}$$

The constraint Equation (27) implies that $\langle {\left(p^{0}\right)}^{2}X^{\alpha }\rangle =\langle p^{0}p^{j}X^{\alpha }\rangle =0$, so Equation (71) simplify to

(72) $$\begin{array}{ccc} \langle p^{0}p^{\mu }p^{\nu }\rangle \beta_{\nu ,\mu } & = & 0 \end{array}$$

(73) $$\begin{array}{ccc} \langle p^{j}p^{\mu }p^{\nu }\rangle \beta_{\nu ,\mu }+\sum_{\alpha =1}^{4}C_{\alpha ,i}\langle p^{i}p^{j}X^{\alpha }\rangle & = & 0 \end{array}$$

(74) $$\begin{array}{ccc} \langle X^{\beta }p^{i}p^{j}\rangle \beta_{i,j}+\sum_{\alpha =1}^{4}\left[{\dot{C}}_{\alpha }\langle X^{\beta }p^{0}X^{\alpha }\rangle +C_{\alpha ,i}\langle X^{\beta }p^{i}X^{\alpha }\rangle \right] & = & 0 \end{array}$$

Computing the averages as defined in Equation (26), we find the full set of equations in the free-streaming regime

(75) $$\begin{array}{ccc} \frac{\dot{t}}{t}+\frac{1}{3}u_{,j}^{j} & = & 0\\ \Theta_{3}^{0}\left(\frac{t_{,i}}{t}+{\dot{u}}^{i}\right)+\frac{1}{5}\Theta_{4}^{1}\Lambda^{\mu \rho ij}C_{1\mu \rho ,j}+\frac{1}{5}A\Lambda^{\mu \rho ij}C_{3\mu \rho ,j} & = & 0\\ \Theta_{4}^{1}\Lambda^{\mu \nu kl}u_{k,l}+\Theta_{5}^{2}\Lambda^{\mu \nu kl}{\dot{C}}_{1kl}+\frac{1}{7}\Theta_{6}^{3}\Lambda^{\mu \nu klmn}C_{2lmn,k}+F\Lambda^{\mu \nu \lambda k}C_{4\lambda ,k} & = & 0\\ \Theta_{6}^{3}\Lambda^{\mu \nu \lambda \rho \sigma k}C_{1\rho \sigma ,k}+\Theta_{7}^{4}\Lambda^{\mu \nu \lambda \rho \sigma \tau }{\dot{C}}_{2\rho \sigma \tau }+G\Lambda^{\mu \nu \lambda \rho \sigma k}C_{3\rho \sigma ,k} & = & 0\\ A\Lambda^{\mu \nu kl}u_{k,l}+\frac{1}{7}G\Lambda^{\mu \nu klmn}C_{2lmn,k}+G\Lambda^{\mu \nu kl}{\dot{C}}_{3kl}+K\Lambda^{\mu \nu lk}C_{4l,k} & = & 0\\ \frac{1}{5}F\Lambda^{\mu \nu \rho k}C_{1\nu \rho ,k}+\frac{1}{5}K\Lambda^{\mu \nu \rho k}C_{3\nu \rho ,k}+M\Delta^{\mu \nu }{\dot{C}}_{4\nu } & = & 0 \end{array}$$

The different coefficients in the equations read

(76) $$\begin{array}{ccc} A & = & \left[\Theta_{5}^{2}-\gamma \Theta_{4}^{1}\right]\\ F & = & \left[D_{2}\Theta_{4}^{2}-\frac{\Theta_{4}^{1}}{\Theta_{3}^{0}}\Theta_{5}^{2}+\Theta_{6}^{3}-D_{3}\Theta_{4}^{1}\right]\\ G & = & \left[\Theta_{7}^{4}-\gamma \Theta_{6}^{3}\right]\\ K & = & \left[D_{2}\Theta_{5}^{3}-\frac{\Theta_{4}^{1}}{\Theta_{3}^{0}}\Theta_{6}^{3}+\Theta_{7}^{4}-D_{3}\Theta_{5}^{2}-\gamma F\right]\\ M & = & \left[D_{2}^{2}\Theta_{3}^{2}+{\left(\frac{\Theta_{4}^{1}}{\Theta_{3}^{0}}\right)}^{2}\Theta_{5}^{2}+\Theta_{7}^{4}+D_{3}^{2}\Theta_{3}^{0}-2D_{2}\frac{\Theta_{4}^{1}}{\Theta_{3}^{0}}\Theta_{4}^{2}-2\frac{\Theta_{4}^{1}}{\Theta_{3}^{0}}\Theta_{6}^{3}+2D_{2}\Theta_{5}^{3}\right.\\ & - & \left.2D_{2}D_{3}\Theta_{3}^{1}+2D_{3}\frac{\Theta_{4}^{1}}{\Theta_{3}^{0}}\Theta_{4}^{1}-2D_{3}\Theta_{5}^{2}\right] \end{array}$$

Observe that these coefficients depend explicitly on the microphysics, due to the presence of the function *F* in (64). In particular the coefficients $D_{2}$, $D_{3}$ and γ, defined in Equations (62), (63) and (66), respectively, vanish in the AW ansatz for the RTA.

## 5. Propagation Speeds

We are now ready to derive the propagation speeds *v* for linearized fluctuations around an equilibrium solution $\beta_{\mu }=\beta_{0\mu }=$ constant, $C^{\alpha }=0$ and α=1−4. These fluctuations represent collective modes of the system of interacting particles. As we show in the following, the propagation speeds *v* may be derived from the dispersion relations obtained from the Fourier transform of Equation (75), which take the form ω=vk. To derive this dispersion relations from the principal terms only is equivalent to considering the full dispersion relations, including dissipative terms, in the limit k→∞.

It is important to highlight that working with the equation system (75) does not make the choice of the function *F* in the collision term irrelevant, because the tensors $X^{\alpha }$ which make up χ depend on it, see Equations (65) and (76). This has stemmed from enforcing the integrability conditions at each order in the Ch-En development, which are essential for the second law of thermodynamics to be fulfilled. Thus, we have written a parameterized 1-pdf general enough to include a second-order Ch-En solution of the collisional theory and work with Equation (75) to investigate the front propagation speeds. We find that the propagation speeds depend on the microphysics through the choice of the function *F*, and for a wide range of choices, are maximized by the AW ansatz F[x]=x.

### 5.1. Relating Front Propagation Speeds to the Dispersion Relations

Regardless of the actual form of the collision term, it is clear that after linearizing around an equilibrium solution and going to the equilibrium rest frame, the equations in (17) take the form

(77) $${\dot{C}}^{\alpha }+N_{\beta }^{j\alpha }C_{,j}^{\beta }+I_{\beta }^{\alpha }C^{\beta }=0$$

where only the last term comes from the collision integral.

Let us seek a solution representing a front (namely, a surface where the fluid variables are continuous but their first derivatives are not) moving into a fluid in equilibrium along the direction $\hat{k}$. The solution depends on time and space only through the variable $\xi =\hat{k}\cdot x-vt$, where *v* is the front velocity. Let the front position be $\xi =\xi_{0}$. At that point, the $C^{\alpha }$’s are continuous, but the lateral ξ− derivatives ${C^{\prime }}_{+}^{\alpha }$ and ${C^{\prime }}_{-}^{\alpha }$ are different ( + and − denote the upstream and downstream parts of the fluid). Therefore, taking the difference of Equation (77) in front of and behind the front, the terms from the collision integral cancel, and we obtain

(78) $$\left[-v\delta_{\beta }^{\alpha }+{\hat{k}}_{j}N_{\beta }^{j\alpha }\right]\left({C^{\prime }}_{+}^{\beta }-{C^{\prime }}_{-}^{\beta }\right)=0$$

with a prime denoting a ξ-derivative.

On the other hand, suppose we seek the dispersion relations which follow from Equation (77). Then, we propose a solution of the form $C^{\alpha }=C_{0}^{\alpha }e^{i\left[k\cdot x-\omega t\right]}$ and obtain

(79) $$\left[\left(-i\omega \right)\delta_{\beta }^{\alpha }+iN_{\beta }^{j\alpha }k_{j}+I_{\beta }^{\alpha }\right]C_{0}^{\beta }=0$$

It is clear that Equation (78) are the same as Equation (79) under the identification ω=vk, $k=k\hat{k}$. We take advantage of this fact and evaluate the propagation speeds from the Fourier transform of system (75). This is not an approximation but rather the definition of the propagation speeds.

### 5.2. SVT Decomposition

As it is well known, the equations of motion can be further decoupled by decomposing the deviations from equilibrium into scalar, vector and tensor quantities. We then write

(80) $$\begin{array}{ccc} u^{i} & = & \vartheta^{i}+\nabla_{i}\vartheta \end{array}$$

(81) $$\begin{array}{ccc} C_{4i} & = & c_{4i}+\nabla_{i}c_{4} \end{array}$$

(82) $$\begin{array}{ccc} C_{1ij} & = & \left(\nabla_{i}\nabla_{j}-\frac{1}{3}\Delta_{ij}\nabla^{2}\right)c_{1}+c_{1i,j}+c_{1j,i}+c_{1ij} \end{array}$$

(83) $$\begin{array}{ccc} C_{3ij} & = & \left(\nabla_{i}\nabla_{j}-\frac{1}{3}\Delta_{ij}\nabla^{2}\right)c_{3}+c_{3i,j}+c_{3j,i}+c_{3ij} \end{array}$$

(84) $$\begin{array}{ccc} C_{2ijk} & = & \left[\nabla_{i}\nabla_{j}\nabla_{k}-\frac{1}{5}\left(\Delta_{ij}\nabla_{k}+\Delta_{ik}\nabla_{j}+\Delta_{jk}\nabla_{i}\right)\nabla^{2}\right]c_{2}\\ & + & \left(\nabla_{i}\nabla_{j}-\frac{1}{5}\Delta_{ij}\nabla^{2}\right)c_{2k}+\left(\nabla_{i}\nabla_{k}-\frac{1}{5}\Delta_{ik}\nabla^{2}\right)c_{2j}+\left(\nabla_{k}\nabla_{j}-\frac{1}{5}\Delta_{kj}\nabla^{2}\right)c_{2i}\\ & + & \nabla_{i}c_{2jk}+\nabla_{j}c_{3ik}+\nabla_{k}c_{2ij}+c_{2ijk} \end{array}$$

Here, quantities with no indexes are scalars, quantities with a single index are vectors (namely, divergenceless), and quantities with more than one index are tensors (divergenceless and traceless).

We shall discuss in some detail the simplest tensor sector and give the results for the vector and scalar ones, which follow the same structure. The homogeneous equation for $c_{2ijk}$ reduces to ${\dot{c}}_{2ijk}=0$, and we do not discuss it.

#### 5.2.1. Tensor Sector

Considering only tensor quantities in Equation (75), we obtain

(85) $$\begin{array}{ccc} \Theta_{5}^{2}{\dot{C}}_{1ij}+\frac{3}{7}\Theta_{6}^{3}\nabla^{2}C_{2ij} & = & 0\\ \Theta_{6}^{3}C_{1ij}+3\Theta_{7}^{4}{\dot{C}}_{2ij}+GC_{3ij} & = & 0\\ G[\frac{3}{7}\nabla^{2}C_{2ij}+{\dot{C}}_{3ij}] & = & 0 \end{array}$$

Therefore, the dispersion relation is derived from the roots of the determinant

(86) $$\det\;\left(\begin{array}{ccc} \Theta_{5}^{2}\left(-i\omega \right) & -\frac{3}{7}\Theta_{6}^{3}k^{2} & 0\\ \Theta_{6}^{3} & 3\Theta_{7}^{4}\left(-i\omega \right) & G\\ 0 & -\frac{3}{7}Gk^{2} & G(-i\omega ) \end{array}\right)=0$$

Explicitly,

(87) $$3G\Theta_{5}^{2}\Theta_{7}^{4}\left(i\omega \right)\left[\omega^{2}-\frac{1}{7}k^{2}\right]=0$$

Observe that although the determinant in Equation (86) becomes singular for the AW RTA, where G=0, the dispersion relation is well defined there and yields the propagation speeds $1/\sqrt{7}$ and 0, independently of the choice of the function *F*, in agreement with [51].

As we now show, the propagation speeds do depend on the choice of the function *F* in the vector and scalar sectors.

#### 5.2.2. Vector Sector

The vector terms in Equation (75) are

(88) $$\begin{array}{ccc} \Theta_{3}^{0}{\dot{\vartheta }}^{i}+\frac{2}{5}\Theta_{4}^{1}\nabla^{2}c_{1i}+\frac{2}{5}A\nabla^{2}c_{3i} & = & 0\\ \nabla_{j}\left[\Theta_{4}^{1}\vartheta_{i}+2\Theta_{5}^{2}{\dot{c}}_{1i}+\frac{24}{35}\Theta_{6}^{3}\nabla^{2}c_{2i}+Fc_{4i}\right] & = & 0\\ \left(\nabla_{j}\nabla_{k}-\frac{1}{5}\Delta_{jk}\nabla^{2}\right)\left[\frac{2}{3}\Theta_{6}^{3}c_{1i}+\Theta_{7}^{4}{\dot{c}}_{2i}+\frac{2}{3}Gc_{3i}\right] & = & 0\\ \nabla_{j}\left[A\vartheta_{i}+\frac{24}{35}G\nabla^{2}c_{2i}+2G{\dot{c}}_{3i}+Kc_{4i}\right] & = & 0\\ \frac{2}{5}F\nabla^{2}c_{1i}+\frac{2}{5}K\nabla^{2}c_{3i}+M{\dot{c}}_{4i} & = & 0 \end{array}$$

wherefrom we obtain the characteristic equation

(89) $$\det\;\left(\begin{array}{ccccc} \Theta_{3}^{0}\left(-i\omega \right) & -\frac{2}{5}k^{2}\Theta_{4}^{1} & 0 & -\frac{2}{5}k^{2}A & 0\\ \Theta_{4}^{1} & 2\Theta_{5}^{2}\left(-i\omega \right) & -\frac{24}{35}k^{2}\Theta_{6}^{3} & 0 & F\\ 0 & \frac{2}{3}\Theta_{6}^{3} & \Theta_{7}^{4}\left(-i\omega \right) & \frac{2}{3}G & 0\\ A & 0 & -\frac{24}{35}Gk^{2} & 2G(-i\omega ) & K\\ 0 & -\frac{2}{5}Fk^{2} & 0 & -\frac{2}{5}Kk^{2} & M(-i\omega ) \end{array}\right)=0$$

#### 5.2.3. Scalar Sector

Keeping only scalar terms in Equation (75) gives

(90) $$\begin{array}{ccc} \dot{t}+\frac{1}{3}\nabla^{2}\vartheta & = & 0\\ \nabla_{i}\left[\Theta_{3}^{0}\left(t+\dot{\vartheta }\right)+\frac{4}{15}\Theta_{4}^{1}\nabla^{2}c_{1}+\frac{4}{15}A\nabla^{2}c_{3}\right] & = & 0\\ 2\left(\nabla_{i}\nabla_{j}-\frac{1}{3}\Delta_{ij}\nabla^{2}\right)\left[\Theta_{4}^{1}\vartheta +\Theta_{5}^{2}{\dot{c}}_{1}+\frac{9}{35}\Theta_{6}^{3}\nabla^{2}c_{2}+Fc_{4}\right] & = & 0\\ 6\left[\nabla_{i}\nabla_{j}\nabla_{k}-\frac{1}{5}\nabla^{2}\Delta_{ij}\nabla_{k}-\frac{1}{5}\nabla^{2}\Delta_{ik}\nabla_{j}-\frac{1}{5}\nabla^{2}\Delta_{jk}\nabla_{i}\right]\left[\Theta_{6}^{3}c_{1}+\Theta_{7}^{4}{\dot{c}}_{2}+Gc_{3}\right] & = & 0\\ 2\left(\nabla_{i}\nabla_{j}-\frac{1}{3}\Delta_{ij}\nabla^{2}\right)\left[A\vartheta +\frac{9}{35}G\nabla^{2}c_{2}+G{\dot{c}}_{3}+Kc_{4}\right] & = & 0\\ \nabla_{i}\left[\frac{4}{15}F\nabla^{2}c_{1}+\frac{4}{15}K\nabla^{2}c_{3}+M{\dot{c}}_{4}\right] & = & 0 \end{array}$$

leading to the dispersion relations

(91) $$\det\;\left(\begin{array}{cccccc} \left(-i\omega \right) & -\frac{1}{3}k^{2} & 0 & 0 & 0 & 0\\ \Theta_{3}^{0} & \Theta_{3}^{0}\left(-i\omega \right) & -\frac{4}{15}\Theta_{4}^{1}k^{2} & 0 & -\frac{4}{15}Ak^{2} & 0\\ 0 & \Theta_{4}^{1} & \Theta_{5}^{2}\left(-i\omega \right) & -\frac{9}{35}\Theta_{6}^{3}k^{2} & 0 & F\\ 0 & 0 & \Theta_{6}^{3} & \Theta_{7}^{4}\left(-i\omega \right) & G & 0\\ 0 & A & 0 & -\frac{9}{35}Gk^{2} & G(-i\omega ) & K\\ 0 & 0 & -\frac{4}{15}Fk^{2} & 0 & -\frac{4}{15}Kk^{2} & M(-i\omega ) \end{array}\right)=0$$

### 5.3. Results

To give some content to the results above, we considered the family of RTA’s where

(92) $$F\left[x\right]=x^{a}$$

for which

(93) $$\Theta_{m}^{n}=\Gamma \left[2+m-an\right]$$

To avoid infrared divergences in the equations of motion, we required a≤2. This family includes the Marle and AW RTAs as particular cases, namely, a=0 and a=1, respectively.

As in the tensor case, the dispersion relations are well defined at a=1, although the matrices in Equations (89) and (91) are singular there. The propagation speeds for a=1 coincide with the values given in [51].

For the vector and scalar sectors, we used the tool “Mathematica” to solve the dispersion relations (89) and (91) and plotted the solutions in the figures below.

**Figure 1:**
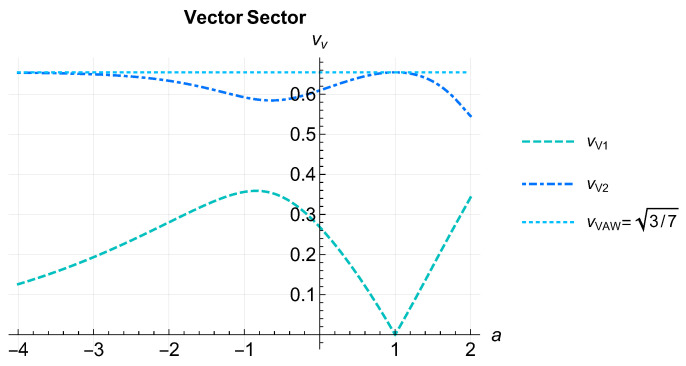
(Color online) Speeds of the two vector modes from Equation (89). The fastest mode (top dot-dashed blue curve) attains the same maximum speed for a=1 and for a→−∞ (top dotted light-blue horizontal line), indicating that the AW value is not exceeded at any value of *a*. Observe that the slowest mode speed (bottom, dashed light-blue curve) is zero for a=1, so we recover the AW case [51] where only one non-null mode exists.

The solutions $v_{V}=\omega /k$ for the vector sector are plotted in Figure 1. The fastest mode (top dot-dashed blue curve) attains the same maximum speed for a=1 and for a→−∞ (top dotted light-blue horizontal line), indicating that the AW value is not exceeded at any value of *a*. Observe that the slowest mode speed (bottom, dashed light-blue curve) is zero for a=1, so we recover the AW case where only one non-null mode exists [51].

**Figure 2:**
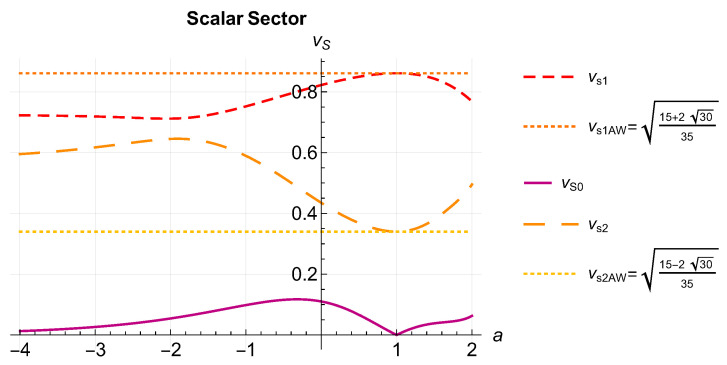
(Color online) Speeds of the three scalar modes from Equation (91). We see that the maximum speed of the fastest mode (top red short-dashed curve) corresponds to the AW solution a=1 (top horizontal orange dotted line). The intermediate speed mode (long-dashed orange curve in the middle of the figure) also attains its minimum value at the AW value a=1 (bottom horizontal yellow dotted line), and the speeds of this mode never exceed the ones of the fastest mode. These two modes are the generalization of the AW modes found elsewhere. The bottom, single-line purple curve corresponds to the speeds of a new, slowest mode, whose velocity for a=1 is zero. Thus, we see that the AW case, for which there are only two propagating modes, is consistently included in our formalism.

The solutions $v_{S}=\omega /k$ for the scalar case are plotted in Figure 2. We see that the maximum speed of the fastest mode (top red short-dashed curve) corresponds to the AW solution a=1 (top horizontal orange dotted line). The intermediate speed mode (long-dashed orange curve in the middle of the figure) attains its minimum value also at the AW value a=1 (bottom horizontal yellow dotted line), and the speed of this mode never exceeds that of the fastest mode. These two modes are the generalization of the AW modes found elsewhere. The bottom, single-line purple curve corresponds to the speeds of a new, slowest mode, whose velocity for a=1 is zero. Thus, we see that the AW case [51], for which there are only two non-null modes, is consistently included in our formalism.

**Figure 3:**
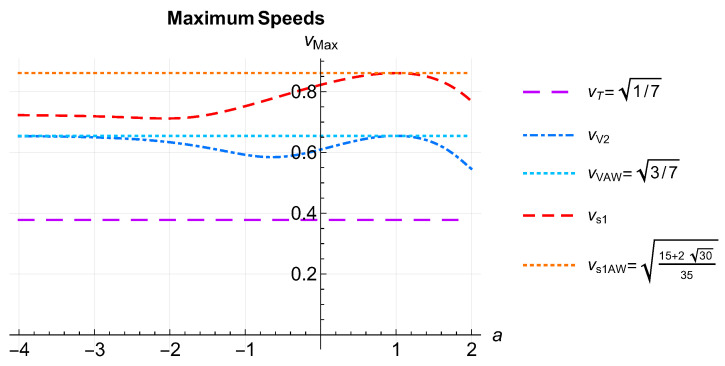
(Color online) Comparison of the maximum propagation speeds of each sector. The top dotted horizontal line corresponds to the AW (a=1) scalar mode speed, short-dashed line immediately below corresponds to the velocities of the scalar fastest mode of our model. The middle dotted horizontal line and dot-dashed middle curve correspond to the vector mode speed for AW (a=1) and to the speeds of our model, respectively. The bottom long-dashed horizontal line is the speed of the tensor mode, which agrees with the AW speed over the entire interval of *a* values considered. They verify $v_{T}<v_{V}<v_{S}$.

Finally, in Figure 3, we plotted the curves that correspond to the speed of the fastest mode of each sector. The top dotted horizontal line corresponds to the AW (a=1) speed, and the short-dashed line immediately below corresponds to the velocities of the scalar fastest mode of our model. The middle dotted horizontal line and dot-dashed middle curve correspond to the vector mode speed for AW (a=1) and to the speeds of our model, respectively. The bottom long-dashed horizontal line is the speed of the tensor mode, which agrees with the AW speed over the entire interval of *a* values considered. They verify $v_{T}<v_{V}<v_{S}$, which curiously is the same order relationship already obtained by Israel and Stewart in ref. [82].

All the propagation speeds are less than the speed of the light. This is a consequence of the causal evolution of the Boltzmann equation, which is something that is broken when performing the Chapman–Enskog procedure (relativistic Navier–Stokes is acausal and unstable) and is recovered by (1-pdf) moment methods such as the one in this paper. For further discussion of causality requirements in relativistic hydrodynamics, see ref. [66].

## 6. Conclusions

Using the Chapman–Enskog expansion, we developed a linearized 1-pdf up to the second order around a local thermal equilibrium. At each order, we enforced the Second Law, so positive entropy production was guaranteed. We then generalized this distribution function by identifying each term in the Ch-En expansion with a product of a momentum-dependent tensor with a parameter that encoded the dissipative properties of the flow. Therefore, the parameterization contained the second-order Ch-En solution as a special case. Using the moments method, we then obtained the linearized equations for scalar, vector and tensor perturbations. We worked with the RTA for the collision integral and considered the relaxation time as a function of the momentum, $F\left[-\beta_{\mu }p^{\mu }\right]$.

The coefficients of the conservation equations depend on the function *F*. They form a family of parameterized theories that describe different phenomenologies depending on the choice of the function *F*. Thus, in Equation (75) there remains information about the microphysics on which the RTA was built. Stated in other words: the choice of *F* is a crucial part of the construction of the RTA.

To analyze a concrete case, we specialized the general equations to the case where $F={\left(\beta_{\mu }p^{\mu }\right)}^{a}$, which included the choice of AW of a=1 [16,17] and that of Marle of a=0 [14,15], widely used in the literature, as particular cases. The choice of a power law, besides being mathematically tractable, has actually been proposed before in the context of relativistic heavy ion collisions from phenomenological considerations [18,23,24,25,26,27,28,29,30,31,32,33,34,35,36,37,38]. For example, the interpolating values 0≤a≤1 were already discussed in refs. [23,24], motivated by its possible application to improve the description of relativistic heavy ion collisions. Here, we included the full range −∞≤a≤2, which is maximal because larger values of *a* leads to infrared divergences in the coefficients of the hydrodynamic equations. Allowing for a negative *a* allowed us to explore distributions heavily biased towards hard modes. The power of the generalized RTA presented here is the ability to reproduce spectral properties of the kinetic equation, most importantly whether zero is an isolated eigenvalue or whether it is embedded in the continuous spectrum. Figure 1 and Figure 2 showed the dependence of the propagation speeds on the choice of *F*, restricted to a power law. Choosing a more general functional form for *F* may be justified by concrete experimental results and/or deduced from the Boltzmann equation [18,19,21,23,37].

The propagation speeds of a theory are fundamental to determine causality and to discuss shock waves, among other effects. The linear conservation equations decouple into three sets, corresponding to the tensor, vector and scalar modes, and we computed the corresponding propagation speeds. The propagation speeds were the phase velocity for plane waves obtained from Equation (75), and as it was emphasized above, they depended on the choice of *F*.

For the given 1-pdf, the number of tensor, vector and scalar modes were two, five and six, respectively.

For the tensor modes, we found the propagation speed was actually independent of *a* and agreed with the AW value [51].

In the vector sector, besides the trivial solution $v_{v}=0$, there were two propagation speeds shown in Figure 1. There, we saw that the fastest propagation speed was bounded above by the AW value [51], which was reached at a=1. The slower mode had $v_{v}=0$ for a=1. Therefore, the number of dynamical vector modes in the AW limit reduced to two, as it must.

For the scalar sector, we obtained three different propagation speeds, as shown in Figure 2. As in the tensor and vector sectors, the fastest mode had maximum velocity at a=1, where we recovered the AW result [51]. For the intermediate mode, we also recovered the lower AW speed when a=1. The speeds of the slowest mode were significantly lower than those of the other two scalar modes and vanished for a=1. Therefore, we recovered the right number of dynamical scalar modes (four) in the AW case.

In Figure 3, we compared the speeds of the three fastest modes. We saw that they satisfied $v_{T}<v_{v}<v_{s}$ throughout the whole range of *a* values [58]. In ref. [82], Israel and Stewart also calculated the propagation speeds for scalar, vector and tensor modes and found the same order relationship obtained in this work.

We expect that including higher orders in the Chapman–Enskog development, besides adding more functions to the 1-pdf parameterization, will produce increasingly higher speeds, which will asymptotically approach the speed of light, as was demonstrated by G. Boillat, T. Ruggieri and I. Müller [55,56,57,58,59].

As stated in Section 3.1, in this work, we worked in the Landau frame throughout. The frame dependence of hydrodynamics, and the ensuing possibility of improving the hydrodynamic description by a judicious choice of frame, are active areas of research [78,79,80]. We intend to provide a deeper analysis of the frame dependence of the results in this paper in forthcoming work.

We believe that the main contributions of this work are as follows: First, the use of the Chapman–Enskog expansion as a template on which to build a parameterized theory with dynamics based on the method of moments. The resulting theory is causal for the full range of values of *a*. Causality is expected because, as we have already said, the theory is built to enforce thermodynamic stability, and it is known that stability, causality and covariance are closely linked [60,61,62,63,64,65,66]. Second, the fastest propagation speeds are found in the AW limit a=1, for all scalar, vector and tensor modes. To the best of our knowledge, the fact that the Anderson–Witting RTA [16,17] produces the fastest propagation speeds is a new result. This has deep implications for the description of strong shocks in relativistic fluids [68], which we expect to elaborate on in a separate contribution.

## Data Availability

No new data were created or analyzed in this study. Data sharing is not applicable to this article.

## Appendix A. Chapman–Enskog Solution

### Appendix A.1. First-Order Ch-En Solution

We obtain the first-order Ch-E solution by setting n=0 in Equations (55) and (56), whereby

(A1) $$Q\left[t^{2}F\left[-\beta_{\nu }p^{\nu }\right]Q\left[\chi_{1}\right]\right]=p^{\mu }u_{\mu }p^{\nu }{\dot{\beta }}_{\nu }^{\left(0\right)}-p^{\rho }\Delta_{\rho }^{\mu }p^{\nu }\beta_{\nu ,\mu }$$

and

(A2) $$0=-\langle p^{\lambda }p^{\rho }p^{\nu }\rangle \left\{u_{\rho }{\dot{\beta }}_{\nu }^{\left(0\right)}-\Delta_{\rho }^{\mu }\beta_{\nu ,\mu }\right\}$$

which we recognize as simply the conservation equation for an ideal energy–momentum tensor. Introducing the functions in Equation (64), then

(A3) $$\langle p^{\lambda }p^{\rho }p^{\nu }\rangle =t^{5}\Theta_{3}^{0}\left[u^{\lambda }u^{\rho }u^{\mu }+\frac{1}{3}\left(u^{\lambda }\Delta^{\nu \rho }+u^{\nu }\Delta^{\rho \lambda }+u^{\rho }\Delta^{\lambda \nu }\right)\right]$$

Decomposing as usual $\beta_{\mu }=u_{\mu }/t$, we obtain

(A4) $$\beta_{\mu ,\nu }=u_{\mu }u_{\nu }\frac{\dot{t}}{t^{2}}-\Delta_{\nu }^{\lambda }u_{\mu }\frac{t_{,\lambda }}{t^{2}}+\frac{1}{t}\left\{-\Delta_{\mu }^{\lambda }u_{\nu }{\dot{u}}_{\lambda }+\frac{1}{2}\sigma_{\mu \nu }+\frac{1}{2}\omega_{\mu \nu }+\frac{1}{3}\Delta_{\mu \nu }u_{,\lambda }^{\lambda }\right\}$$

where $\sigma_{\mu \nu }$ is the shear tensor Equation (58) and

(A5) $$\omega_{\mu \nu }=\left[\Delta_{\mu }^{\rho }\Delta_{\nu }^{\sigma }-\Delta_{\nu }^{\rho }\Delta_{\mu }^{\sigma }\right]u_{\rho ,\sigma }$$

(A6) $${\dot{\beta }}_{\mu }=-u_{\mu }\frac{\dot{t}}{t^{2}}+\frac{1}{t}{\dot{u}}_{\mu }$$

This yields Equation (A2) in the familiar form

(A7) $$\begin{array}{ccc} \frac{{\dot{t}}^{0}}{t} & = & -\frac{1}{3}u_{,\nu }^{\nu } \end{array}$$

(A8) $$\begin{array}{ccc} {\dot{u}}^{\mu \left(0\right)} & = & -\Delta^{\mu \nu }\frac{t_{,\nu }}{t} \end{array}$$

We use these equations to eliminate the time derivatives from Equation (A1), leading to

(A9) $$Q\left[t^{2}FQ\left[\chi_{1}\right]\right]=-\frac{p^{\mu }p^{\rho }}{2t}\sigma_{\mu \rho }$$

The solution to Equation (A9) is

(A10) $$FQ\left[\chi_{1}\right]=-\frac{p^{\mu }p^{\rho }}{2t^{3}}\sigma_{\mu \rho }-c_{\mu }p^{\mu }$$

We find a new integrability condition

(A11) $$0=\langle \frac{p^{\lambda }p^{\mu }p^{\rho }}{F}\rangle \frac{\sigma_{\mu \rho }}{2t^{3}}-c_{\mu }\langle \frac{p^{\lambda }p^{\mu }}{F}\rangle$$

The first term is zero, and therefore the homogeneous solution vanishes. Now, again,

(A12) $$\chi_{1}=-\frac{p^{\mu }p^{\rho }}{2t^{3}F}\sigma_{\mu \rho }-c_{\mu }^{\prime }p^{\mu }$$

and imposing the constraint Equation (27), we see that again, $c_{\mu }^{\prime }=0$, whence Equation (57).

### Appendix A.2. Second-Order Ch-En Solution

We obtain the second-order Ch-E solution setting n=1 in Equations (55) and (56). Let us start from the integrability condition

(A13) $$0=-u_{\mu }\left\{\langle p^{\lambda }p^{\mu }p^{\nu }\rangle {\dot{\beta }}_{\nu }^{\left(1\right)}+\langle p^{\lambda }p^{\mu }{\dot{\chi }}_{1}^{\left(0\right)}\rangle \right\}+\Delta_{\rho }^{\mu }\langle p^{\lambda }p^{\rho }\chi_{1,\mu }\rangle$$

Under the linearized approximation,

(A14) $$\begin{array}{ccc} \chi_{1,\mu } & = & -\frac{p^{\rho }p^{\sigma }}{2t^{3}F}\sigma_{\rho \sigma ,\mu }\\ & = & -\frac{p^{\rho }p^{\sigma }}{2t^{3}F}\Lambda_{\rho \sigma }^{\lambda \tau }u_{\lambda ,\tau \mu } \end{array}$$

in particular,

(A15) $$\begin{array}{ccc} {\dot{\chi }}_{1}^{\left(0\right)} & = & -\frac{p^{\rho }p^{\sigma }}{2t^{3}F}\Lambda_{\rho \sigma }^{\lambda \tau }{\dot{u}}_{\lambda ,\tau }^{\left(0\right)}\\ & = & \frac{p^{\rho }p^{\sigma }}{2t^{3}F}\Lambda_{\rho \sigma }^{\lambda \tau }\frac{t_{,\lambda \tau }}{t} \end{array}$$

To evaluate the integrability condition Equation (A13), we compute the mean values $\langle p^{\lambda }p^{\mu }p^{\nu }\rangle$ and $\langle p^{\lambda }p^{\mu }p^{\nu }p^{\rho }/F\rangle$, obtaining

(A16) $$\begin{array}{ccc} & - & u_{\mu }\langle p^{\lambda }p^{\mu }p^{\nu }\rangle {\dot{\beta }}_{\nu }^{\left(1\right)}=t^{4}\Theta_{3}^{0}\left\{u^{\lambda }\frac{{\dot{t}}^{1}}{t}+\frac{1}{3}{\dot{u}}^{\left(1\right)\lambda }\right\}\\ & - & u_{\mu }\langle p^{\lambda }p^{\mu }{\dot{\chi }}_{1}^{\left(0\right)}\rangle =0\\ & & \Delta_{\rho }^{\mu }\langle p^{\lambda }p^{\rho }\chi_{1,\mu }\rangle =\frac{-1}{15}t^{3}\Theta_{4}^{1}\sigma_{,\rho }^{\lambda \rho } \end{array}$$

and we obtain

(A17) $$\begin{array}{ccc} {\dot{t}}^{1} & = & 0\\ {\dot{u}}^{\left(1\right)\lambda } & = & \frac{1}{5t}\frac{\Theta_{4}^{1}}{\Theta_{3}^{0}}\sigma_{,\rho }^{\lambda \rho } \end{array}$$

We now turn to Equation (55)

(A18) $$\begin{array}{ccc} & & Q\left[t^{2}FQ\left[\chi_{2}\right]\right]=p^{\mu }u_{\mu }\left\{p^{\nu }{\dot{\beta }}_{\nu }^{\left(1\right)}-{\dot{\chi }}_{1}^{\left(0\right)}\right\}-p^{\rho }\Delta_{\rho }^{\mu }\chi_{1,\mu }\\ & = & p^{\mu }u_{\mu }\left\{p^{\nu }\frac{1}{5t^{2}}\frac{\Theta_{4}^{1}}{\Theta_{3}^{0}}\sigma_{\nu ,\rho }^{\rho }-\frac{p^{\rho }p^{\nu }}{2t^{3}F}\Lambda_{\rho \nu }^{\lambda \tau }\frac{t_{,\lambda \tau }}{t}\right\}+p^{\rho }\Delta_{\rho }^{\lambda }\frac{p^{\nu }p^{\mu }}{2t^{3}F}\sigma_{\nu \mu ,\lambda } \end{array}$$

A first integration yields

(A19) $$t^{2}FQ\left[\chi_{2}\right]=p^{\mu }u_{\mu }\left\{p^{\nu }\frac{1}{5t^{2}}\frac{\Theta_{4}^{1}}{\Theta_{3}^{0}}\sigma_{\nu ,\rho }^{\rho }-\frac{p^{\rho }p^{\nu }}{2t^{3}F}\Lambda_{\rho \nu }^{\lambda \tau }\frac{t_{,\lambda \tau }}{t}\right\}+p^{\rho }\Delta_{\rho }^{\lambda }\frac{p^{\nu }p^{\mu }}{2t^{3}F}\sigma_{\nu \mu ,\lambda }-d_{\mu }p^{\mu }$$

where the $d_{\mu }$’s are integration constants. We thereby find the integrability condition

(A20) $$0=\langle \frac{p^{\lambda }}{t^{2}F}\left\{p^{\mu }u_{\mu }\left[p^{\nu }\frac{1}{5t^{2}}\frac{\Theta_{4}^{1}}{\Theta_{3}^{0}}\sigma_{\nu ,\rho }^{\rho }-\frac{p^{\rho }p^{\nu }}{2t^{3}F}\Lambda_{\rho \nu }^{\lambda \tau }\frac{t_{,\lambda \tau }}{t}\right]+p^{\rho }\Delta_{\rho }^{\tau }\frac{p^{\nu }p^{\mu }}{2t^{3}F}\sigma_{\nu \mu ,\tau }-d_{\mu }p^{\mu }\right\}\rangle$$

which reduces to

(A21) $$0=-\frac{1}{15}\left[\Theta_{4}^{2}-\frac{\Theta_{4}^{1}}{\Theta_{3}^{0}}\Theta_{3}^{1}\right]\sigma_{,\rho }^{\lambda \rho }+td_{\mu }\Theta_{2}^{1}\left[u^{\lambda }u^{\mu }+\frac{1}{3}\Delta^{\mu \nu }\right]$$

or else

(A22) $$d_{\mu }=-\frac{1}{5t}D_{2}\sigma_{\mu ,\rho }^{\rho }$$

where $D_{2}$ was defined in Equation (62) We thus arrive at the preliminary result

(A23) $$\chi_{2}=\frac{1}{t^{2}F}\left\{p^{\mu }u_{\mu }\left[p^{\nu }\frac{1}{5t^{2}}\frac{\Theta_{4}^{1}}{\Theta_{3}^{0}}\sigma_{\nu ,\rho }^{\rho }-\frac{p^{\rho }p^{\nu }}{2t^{3}F}\Lambda_{\rho \nu }^{\lambda \tau }\frac{t_{,\lambda \tau }}{t}\right]+p^{\rho }\Delta_{\rho }^{\tau }\frac{p^{\nu }p^{\mu }}{2t^{3}F}\sigma_{\nu \mu ,\tau }-d_{\mu }p^{\mu }\right\}-d_{\mu }^{\prime }p^{\mu }$$

with a new set of integration constants $d_{\mu }^{\prime }$. To fix $d_{\mu }^{\prime }$, we must enforce the constraint Equation (27). Introducing the tensor Σ from Equation (61), we obtain

(A24) $$\begin{array}{ccc} \chi_{2} & = & \frac{1}{t^{2}}\Sigma_{\nu \mu \rho }\frac{p^{\nu }p^{\mu }p^{\rho }}{2t^{3}F^{2}}-\frac{1}{t^{3}}u_{\mu }t_{,\lambda \tau }\Lambda_{\rho \nu }^{\lambda \tau }\frac{p^{\nu }p^{\mu }p^{\rho }}{2t^{3}F^{2}}\\ & + & \frac{1}{5t^{3}}\sigma_{\nu ,\rho }^{\rho }p^{\nu }\left[\frac{D_{2}}{F}+\frac{\Theta_{4}^{1}}{\Theta_{3}^{0}}\frac{p^{\mu }u_{\mu }}{tF}+{\left(\frac{p^{\mu }u_{\mu }}{tF}\right)}^{2}\right]-d_{\mu }^{\prime }p^{\mu } \end{array}$$

Enforcing the constraint Equation (27) yields Equation (60).
